# CSP-UNet: A Lightweight Network for Hand X-Ray Image Segmentation

**DOI:** 10.3390/jimaging12090410

**Published:** 2026-09-01

**Authors:** Hai Wang, Jiale Gu, Junhao Wen, Chunlai Yang

**Affiliations:** 1 School of Mechanical Engineering, Anhui Polytechnic University, Wuhu 241000, China; wanghai@ahpu.edu.cn (H.W.); 18862228705@163.com (J.G.); wjh715wjh@outlook.com (J.W.); 2 Anhui Key Laboratory of Advanced Numerical Control & Servo Technology, Wuhu 241000, China

**Keywords:** medical image segmentation, hand X-rays, deep learning, lightweight, data augmentation

## Abstract

Hand X-ray image segmentation is an important step in automated radiographic image analysis. However, conventional U-shaped segmentation networks often have relatively high model complexity, while variations in grayscale distributions across hand X-ray images may affect segmentation performance. To address these issues, this study proposes a lightweight hand X-ray image segmentation network, CSP-UNet (Cross-Stage Partial U-Net). The network integrates cross-stage partial feature processing into the U-Net encoder–decoder framework to reduce redundant feature computation and the number of model parameters while preserving effective feature representation. In addition, an adaptive Gaussian histogram-matching strategy is employed to reduce variations in grayscale distributions across X-ray images. CSP-UNet was evaluated on a dataset comprising 2000 hand X-ray images and compared with Classic U-Net, Res-UNet, Attention U-Net, and Swin U-Net. Experimental results show that CSP-UNet maintained comparable segmentation performance, achieving a Dice coefficient of 0.9927, PA of 0.9839, MPA of 0.9810, and mIoU of 0.9542, while requiring only 20.01 M parameters. Compared with Classic U-Net, CSP-UNet maintained a comparable Dice coefficient (0.9927 vs. 0.9910) while reducing the parameter count from 69.1 M to 20.01 M, corresponding to a reduction of approximately 71.04%. These results indicate that CSP-UNet maintains comparable segmentation performance while substantially reducing model complexity, offering a favorable trade-off between segmentation performance and model size for hand X-ray image segmentation.

## 1. Introduction

Medical image segmentation plays an important role in clinical diagnosis, treatment planning, lesion assessment, quantitative image analysis, and other computer-assisted medical applications [1,2]. By separating anatomical structures or regions of interest from surrounding tissues, image segmentation provides essential information for subsequent image analysis and clinical assessment.

Medical image segmentation methods can generally be divided into traditional image processing methods and deep learning-based methods. Traditional methods mainly rely on gray-level thresholding, region-based processing, edge detection, clustering, and mathematical morphology. Threshold-based methods can achieve effective segmentation when the gray-level distributions of the foreground and background are clearly separable [3]. However, their performance may deteriorate when images contain noise, intensity inhomogeneity, similar gray levels among different tissues, or indistinct boundaries. These factors are common challenges in medical image segmentation and may reduce the reliability of traditional methods that primarily depend on manually defined image features or rules [1,2,3]. Therefore, deep learning-based methods have increasingly been adopted to improve feature representation in complex medical images [2].

With the rapid development of deep learning, convolutional neural networks (CNNs) have become widely used in medical image segmentation because of their ability to learn hierarchical feature representations directly from data [2]. U-Net [4] introduced a symmetric encoder–decoder architecture with skip connections to combine high-level semantic information with low-level spatial details and has become a representative framework for biomedical image segmentation. Subsequent variants further improved feature representation through residual learning, attention mechanisms, and multiscale feature fusion. For example, Attention U-Net [5] introduced attention gates to suppress irrelevant regions and emphasize task-relevant anatomical structures. More recently, Transformer-based architectures have been incorporated into U-shaped networks to enhance long-range dependency modeling and global contextual representation [6]. Representative methods include Swin-Unet [7] and TransUNet [8], which combine hierarchical or hybrid Transformer-based feature modeling with encoder–decoder segmentation frameworks.

Recent studies have increasingly focused on lightweight medical image segmentation to reduce model complexity while maintaining segmentation performance. Ding et al. [9] proposed a lightweight U-Net with fewer downsampling and upsampling stages and multiscale convolutions for pediatric hand X-ray segmentation, demonstrating the feasibility of compact U-shaped architectures for this task. Liao et al. [10] subsequently proposed LightM-UNet, which incorporates Mamba-based sequence modeling into a U-shaped architecture to capture long-range dependencies with reduced model complexity. More recently, Hossain and Brattain [11] developed BiSe-UNet by combining spatial and contextual feature extraction with depthwise-separable decoding for efficient medical image segmentation. These studies demonstrate the growing importance of balancing segmentation performance and computational efficiency. Nevertheless, reducing model complexity without sacrificing spatial details and feature representation remains challenging, particularly when fine anatomical boundaries and narrow inter-finger regions need to be preserved in hand X-ray segmentation [9].

Beyond lightweight architectures, several representative encoder–decoder networks have contributed to the development of medical image segmentation. SegNet [12] introduced an encoder–decoder framework with pooling-index-based upsampling, while 3D U-Net [13] and V-Net [14] extended U-shaped architectures to volumetric medical image segmentation. Residual learning [15] has also been incorporated into U-shaped networks to improve feature propagation and network optimization, leading to variants such as Res-UNet [16]. UNet++ [17] further redesigned skip connections through nested and dense pathways to reduce the semantic gap between encoder and decoder features. In addition, nnU-Net [18] demonstrated that systematic adaptation of network configurations and training strategies can provide strong performance across a wide range of biomedical segmentation tasks. These studies further highlight the importance of balancing feature representation, network complexity, and task-specific design in medical image segmentation.

The main contributions of this study are summarized as follows:

(1) Gaussian histogram-matching strategy combined with K-means clustering is developed to construct representative matching templates according to image brightness, thereby reducing grayscale distribution differences among hand X-ray images.
(2) A lightweight CSP-UNet is developed by introducing CSPDX and CSP2 blocks into the encoder and decoder, respectively. The cross-stage partial design reduces redundant feature computation and model parameters while retaining effective feature representation for hand X-ray segmentation.
(3) A further simplified variant, SCSP-UNet, is constructed by reducing the number of downsampling and upsampling stages to investigate the trade-off between segmentation performance and model complexity.

## 2. Materials and Methods

**Figure 1:**
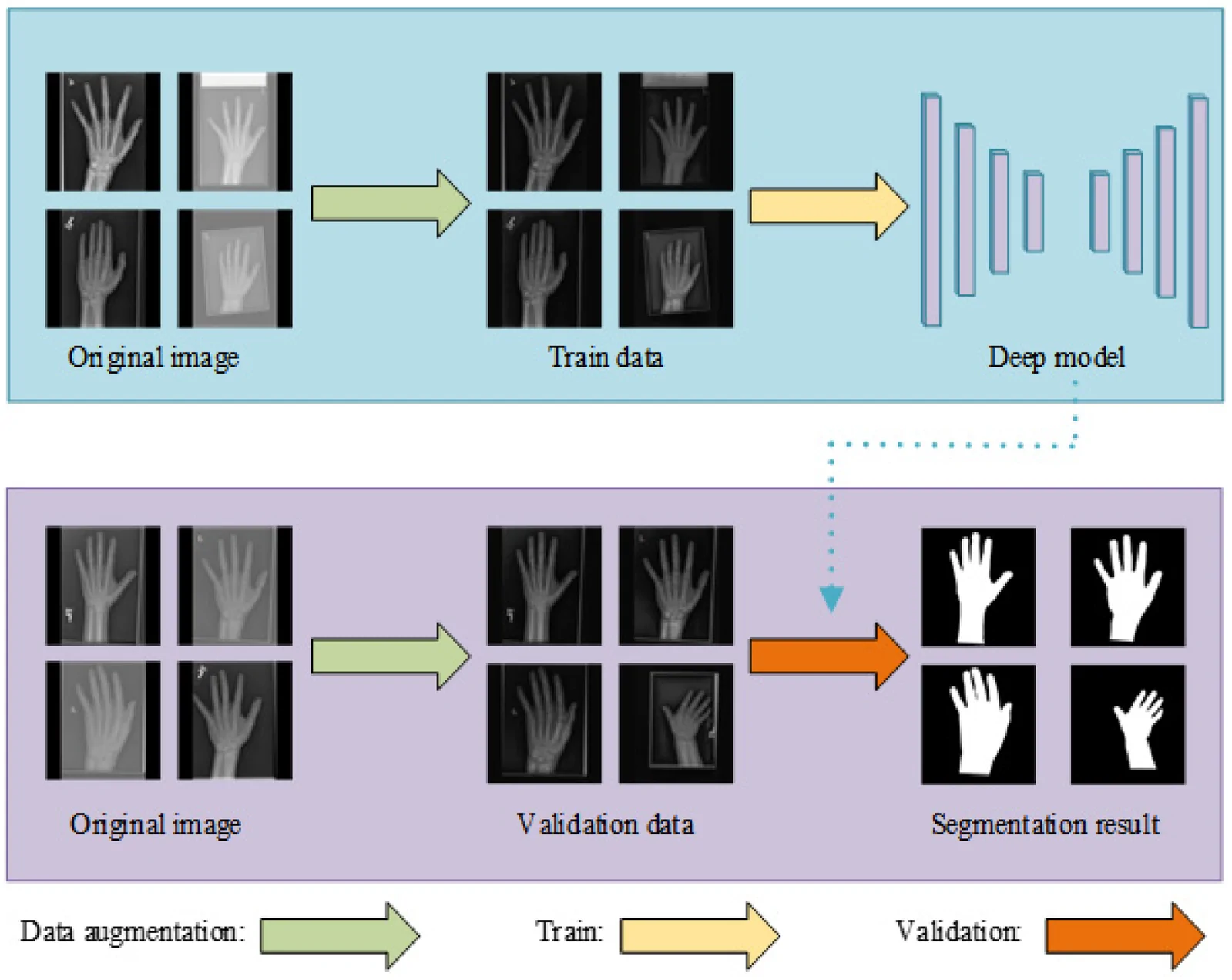
Experimental flow chart.

The architecture of CSP-UNet is shown in Figure 1. The images in the dataset are processed using adaptive Gaussian histogram matching before training and then fed into an encoder containing cross-stage partial dense networks (CSPDX) to obtain feature maps at different levels. Subsequently, decoding is performed using cross-stage double convolutional (CSP2) structures. The output generated by the decoder is used to reconstruct the segmentation results of hand X-ray images. Finally, a lightweight SCSP-UNet is proposed by reducing the numbers of downsampling and upsampling operations in CSP-UNet, and its performance is compared with baseline networks.

### 2.1. Dataset

For the experimental analysis, a total of 2000 hand X-ray images were used in this study. The dataset was collected from two sources. First, 1000 hand X-ray images were retrospectively obtained from the radiology department of a local hospital in Wuhu, China. Each image corresponded to a different patient. All patient-identifying information was removed before analysis, and only anonymized radiographic data were used in this study. The study qualified for exemption from ethical review in accordance with Article 32 of the Measures for Ethical Review of Life Science and Medical Research Involving Human Subjects issued by the National Health Commission of China in 2023. Second, 1000 hand X-ray images were randomly selected from the publicly available RSNA Pediatric Bone Age Machine Learning Challenge dataset [19], which contains hand radiographs collected for bone-age assessment.

The two subsets were combined to construct a dataset containing 2000 images. The hand-region segmentation masks were manually delineated according to the visible hand boundaries in the X-ray images. The annotations were subsequently reviewed and corrected before model training to ensure annotation consistency. Because the present task focused on binary segmentation of the visible hand region rather than the delineation of pathological or diagnostically ambiguous structures, clinicians or radiologists were not involved in the annotation process. All images and their corresponding segmentation masks were then manually inspected and standardized before model training. The complete dataset was randomly divided into training, validation, and test sets at a ratio of 8:1:1, corresponding to 1600, 200, and 200 images, respectively. The model performance reported in this study was based on this fixed dataset split. Images from the same patient were assigned to only one subset to avoid data leakage.

### 2.2. Data Augmentation

**Figure 2:**
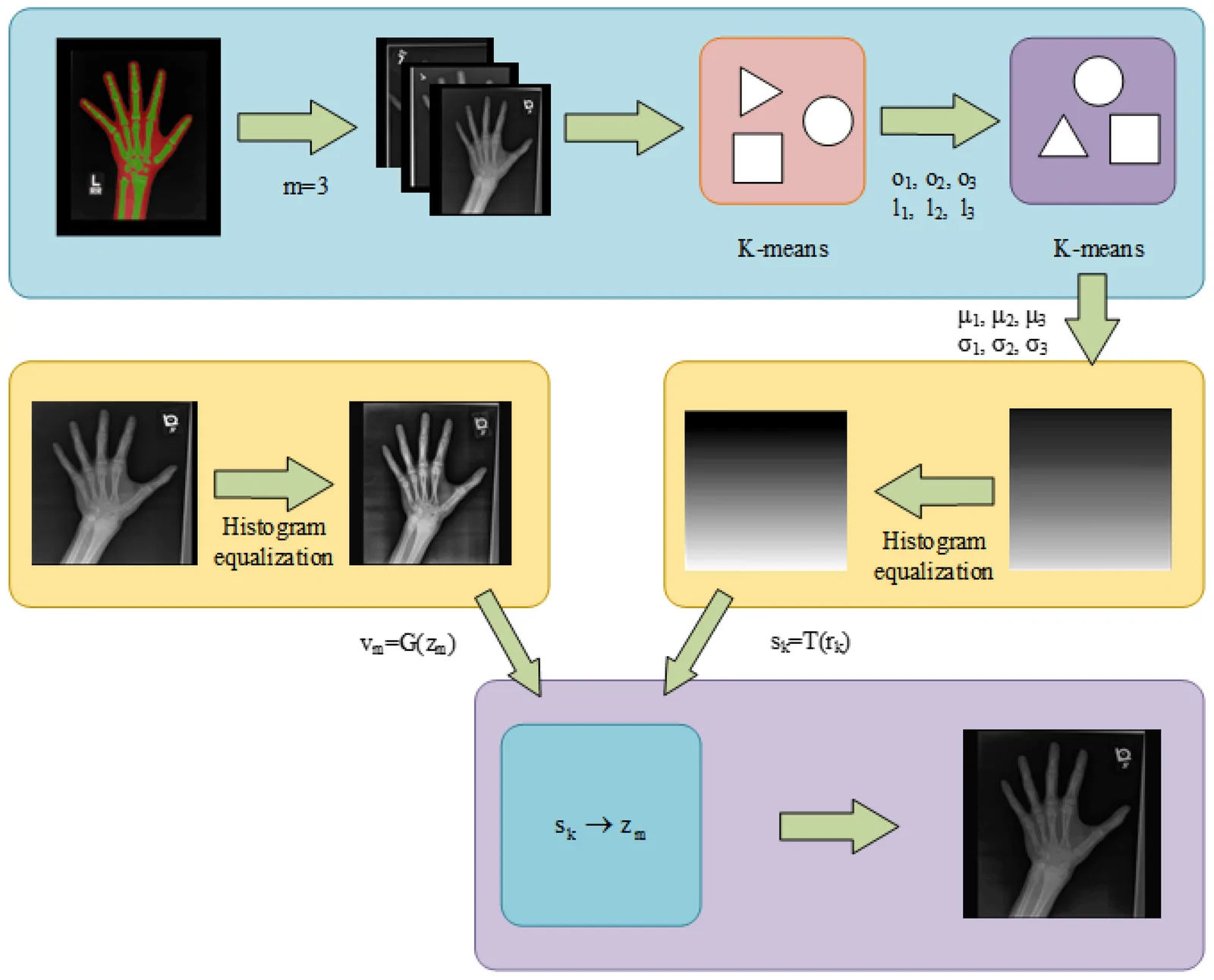
Adaptive Gaussian histogram matching flowchart.

To reduce grayscale intensity variations among hand X-ray images, an adaptive Gaussian histogram-matching method was applied before network training. The method used K-means clustering only to group images according to their grayscale characteristics and to construct representative histogram-matching templates for different image groups. K-means clustering is not used to determine the hand region or generate segmentation masks. Each image is subsequently adjusted according to the corresponding template. The overall procedure is shown in Figure 2.

**Figure 3:**
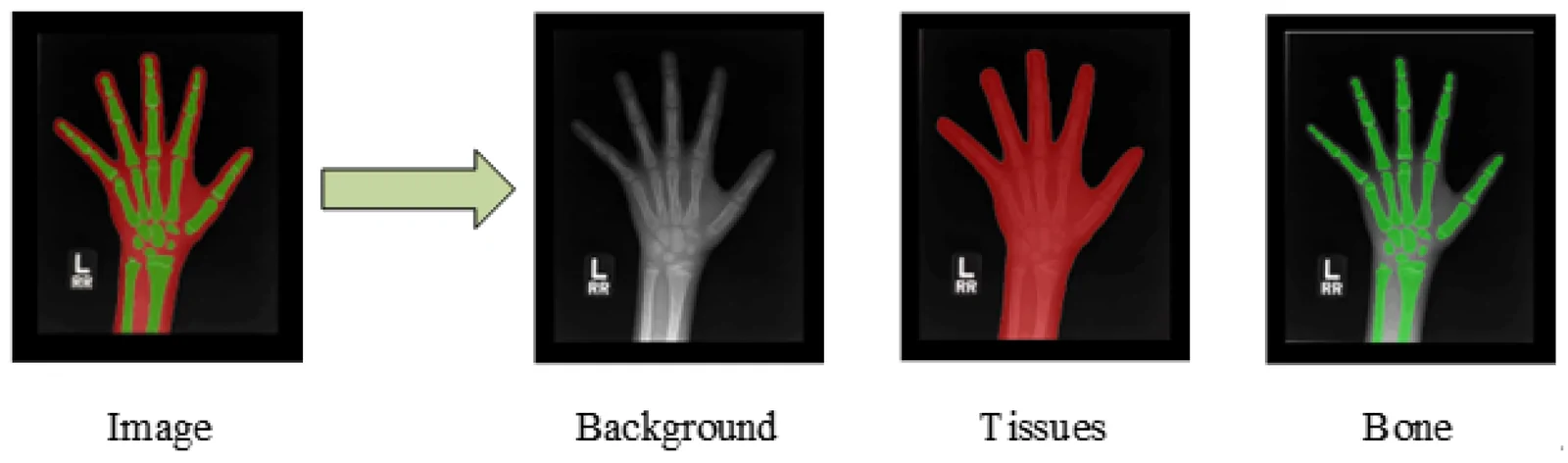
Hand image composition.

(1) As shown in Figure 3, the bone, subcutaneous soft tissue, and background regions in hand X-ray images exhibit different grayscale characteristics. Based on these grayscale differences, the corresponding intensity distributions are described by Equations (1) and (2).

(1) $$f_{n}(n)=\left\lfloor \frac{10}{\sqrt{2\pi }}e^{-\frac{{\left(n-\mu_{m}\right)}^{2}}{2\sigma_{m}^{2}}}\right\rfloor \;\;\;\;\;\;\;\;\;\;\;\;\;\;\;\;\;\;\;\;\;\;\;\;\;\;m=1,2,3$$

(2) $$s=\sum_{n=0}^{a}f_{1}(n)+\sum_{a}^{b}f_{2}(n)+\sum_{b}^{255}f_{3}(n)$$

where n denotes the grayscale level ranging from 0 to 255; m = 1, 2, 3 represents the three Gaussian components; $\mu_{m}$ and $\sigma_{m}$ denote the mean and standard deviation of the m-th Gaussian component, respectively; $f_{n}(n)$ represents the corresponding Gaussian function at grayscale level n; and s denotes the total number of pixels in a single image.

**Figure 4:**
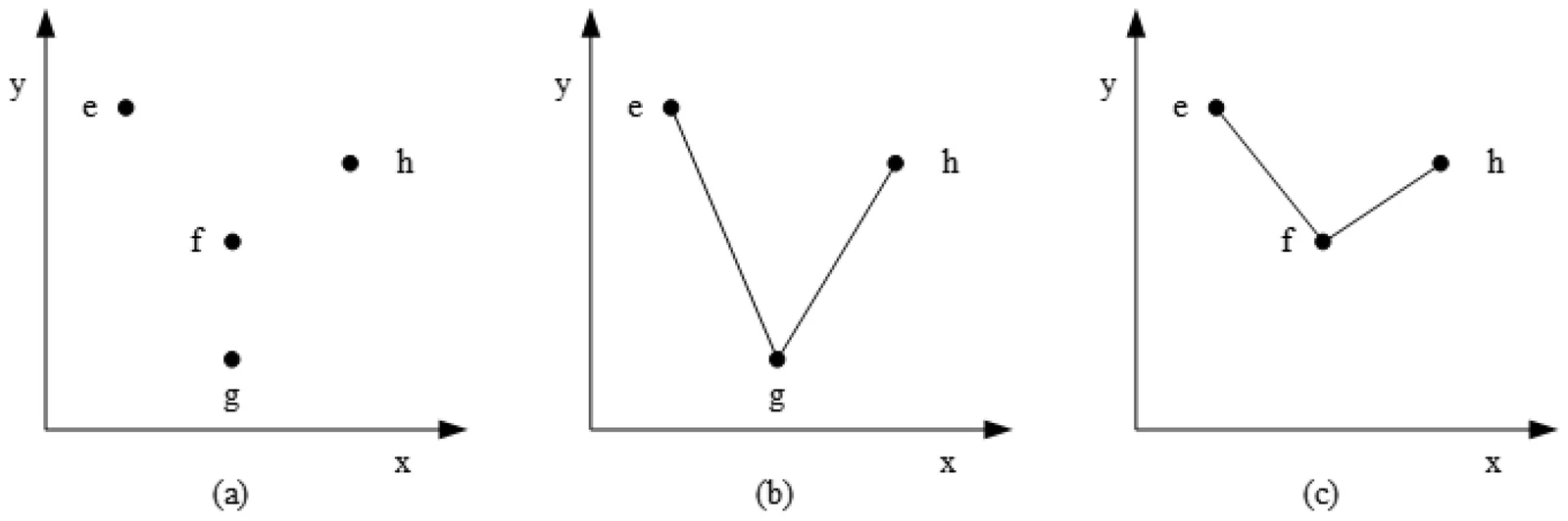
Value map at the junction; (**a**) Maximum and minimum, (**b**) Tip transition, (**c**) Smooth transition.

Where a corresponds to the grayscale level at which the difference between $f_{1}(n)$ and $f_{2}(n)$ is minimized within the interval from μ to 3μ, and b is similarly determined from $f_{2}(n)$ and $f_{3}(n)$. To avoid the tipping phenomenon at the junction, as shown in Figure 4, the values of $f_{m}(n)$ and $f_{m+1}(n)$ at a or b are used. To avoid sharpness issues in images, a large value should be set at the junction of the two functions. That is, when n=a, $f\left(a\right)=max\left(f_{1}\left(a\right);f_{2}\left(a\right)\right)$, and when n=b, $f\left(b\right)=max\left(f_{2}\left(b\right);f_{3}\left(b\right)\right)$.

(2) According to the three Gaussian components defined in Equation (1), K-means clustering was applied to the grayscale intensities of each X-ray image to obtain three intensity clusters. The clustering was used only to estimate the grayscale-distribution parameters required for constructing the histogram-matching template; the resulting intensity clusters were not used as hand-region segmentation masks, and the ratio $o_{1}$, $o_{2}$, and $o_{3}$ for each cluster center and the average Euclidean distance $l_{1}$, $l_{2}$, and $l_{3}$ for different groups in a cluster to the cluster center of the corresponding category were obtained. Then, k-means clustering for three categories was carried out based on the cluster centers and the average distance between cluster pairs for all images in the training dataset. The cluster centers $\mu_{1}$, $\mu_{2}$, and $\mu_{3}$ were obtained. The cluster centers of the Euclidean distance were $L_{1}$, $L_{2}$, and $L_{3}$. Then, the distance cluster centers were adjusted by $\sigma_{1}=L_{1}/\min(L_{1};L_{2};L_{3})$, $\sigma_{2}=L_{2}/\min(L_{1};L_{2};L_{3})$, and $\sigma_{3}=L_{3}/\min(L_{1};L_{2};L_{3})$. Finally, the template histogram was generated according to a template histogram function.

(3) A histogram-matched template map was generated based on the template histogram in (2).

(4) Histogram equalization was performed on the template images, the gray value $s_{k}$ was obtained after the equalization transformation of the template image histogram, and the result was rounded to an integer in the range of $\left[0;L-1\right]$, as shown in Formula (3):

(3) $$s_{k}=T(r_{k})=(L-1)\sum_{i=0}^{k}P_{r}(r_{i})=\frac{\left(L-1\right)}{MN}\sum_{i=0}^{k}n_{i}\;\;\;\;\;\;\;\;\;\;\;\;\;k=0,1,\cdots ,L-1$$

In formula 3, MN is the product of the number of rows M and the number of columns N of the X-ray film pixels in the dataset, and L is the upper limit of the digital interval in which the grayscale of the image is located, that is, the possible gray level series in the image. $n_{i}$ represents the number of pixels with a gray value equal to i. $s_{k}$ is the gray value of the input image, $r_{k}$ is the gray value mapped to the target image after conversion, and $T(r_{k})$ represents the transformation function that maps the gray input value $r_{k}$ to $s_{k}$.

(5) Histogram equalization is performed on the input images, the gray value vm is obtained after the histogram equalization transformation of the input image, the result is rounded to an integer in the range of $\left[0;L-1\right]$, and the $v_{m}$ values are summed. The result is stored in a table, and this process is expressed as shown in Formula (4):

(4) $$v_{m}=G(z_{m})=(L-1)\sum_{j=0}^{m}P_{z}(z_{j})=\frac{\left(L-1\right)}{MN}\sum_{j=0}^{m}n_{j}\;\;\;\;\;\;\;\;\;\;\;\;\;\;\;\;\;\;\;\;m=0,1,\cdots ,L-1$$

In Formula (4), MN is the product of the number of rows M and the number of columns N in the template image, L is the upper limit of the digital interval in which the grayscale of the image is located, that is, the number of possible grayscale levels in the image, and $n_{j}$ represents the pixels with a grayscale value of j. $v_{m}$ is the gray value of the input gray value $z_{m}$ mapped to the target image after transformation, and $G(z_{m})$ represents the transformation function that maps the input gray value $z_{m}$ to $v_{m}$.

(6) For each value $s_{k}$, k=1,2,⋯,L−1, and the $v_{m}$ value stored in (4) is used to find the corresponding $z_{m}$ value so that $G(z_{m})$ is as close to $s_{k}$ as possible. The resulting values from s to z are stored for mapping. When there is more than one $z_{m}$ value satisfying a given $s_{k}$ (that is, when the mapping is not unique), the smallest value is selected, as is convention.

(7) The input image is used for histogram equalization before mapping each equalized pixel value $s_{k}$ in the image to the corresponding $z_{m}$ value in the histogram-specified image.

### 2.3. CSP-UNet

**Figure 5:**
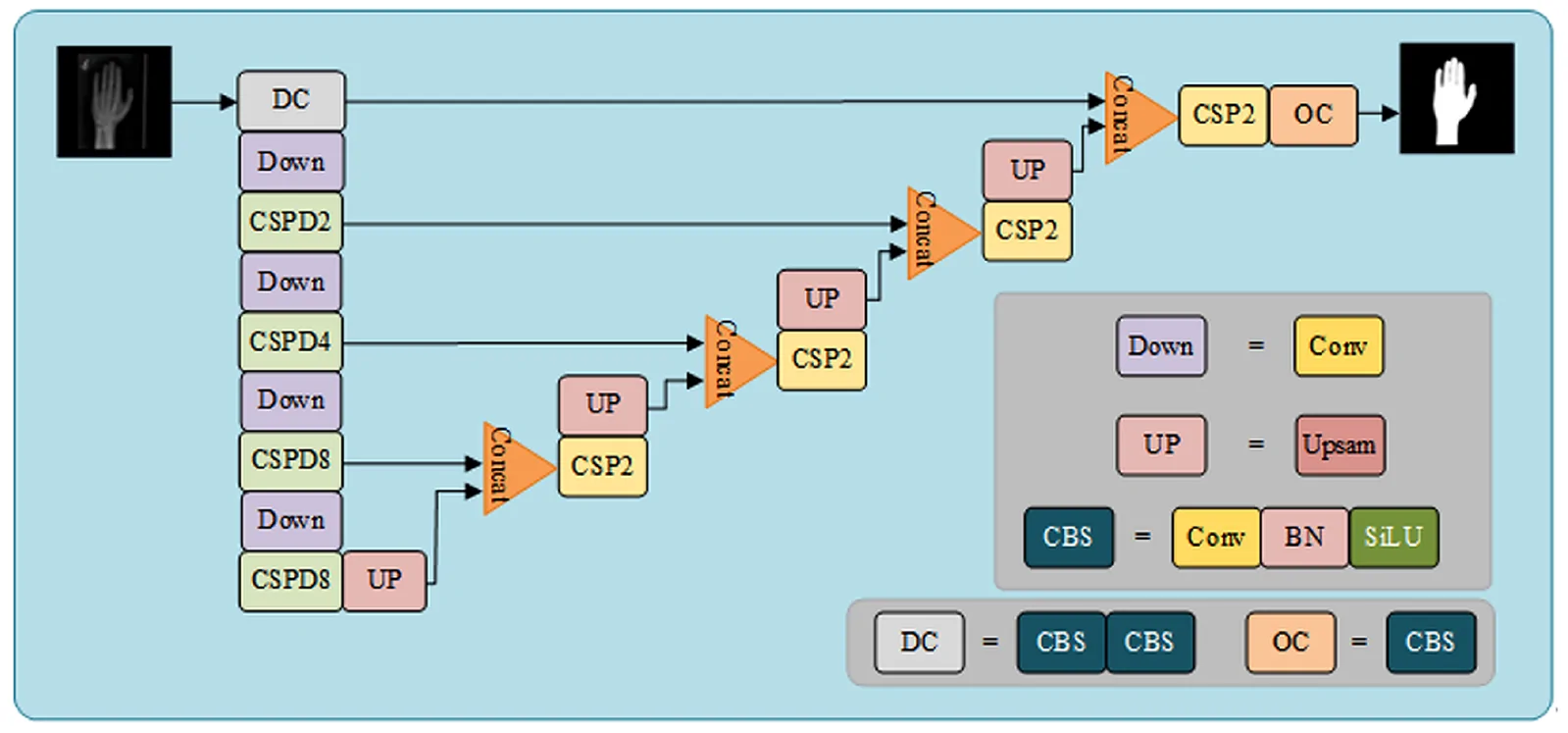
CSP-UNet structure diagram.

CSP-UNet is designed based on the classic U-Net framework [20], with the CSP structure integrated into the encoder and decoder to reduce the number of parameters. The encoder and decoder of CSP-UNet incorporate CSPDX and CSP2 blocks, respectively, as shown in Figure 5. The encoder–decoder structure of the network supports inputs and outputs with the same size, and the entire network is trained in an end-to-end manner. In the encoder, the CSPDX block replaces the double convolution block in the classic U-Net encoder for image feature extraction, enhancing the feature extraction capability of the network. To construct a lightweight network, the CSP2 block is applied in the decoder to recover feature information at different resolutions and spatially fuse and reconstruct features from different layers, thereby achieving semantic segmentation of hand X-ray images.

In Figure 5, Conv is convolution, BN is normalization, SiLU is the activation function, Down is downsampling, Up is upsampling, CBS is the convolution module, CSPDX is a CSP structure containing X convolution blocks, and CSP2 is a double convolutional CSP structure. Detailed convolution parameters are shown in Table 1.

#### 2.3.1. Encoder with a CSPDX Structure

**Figure 6:**
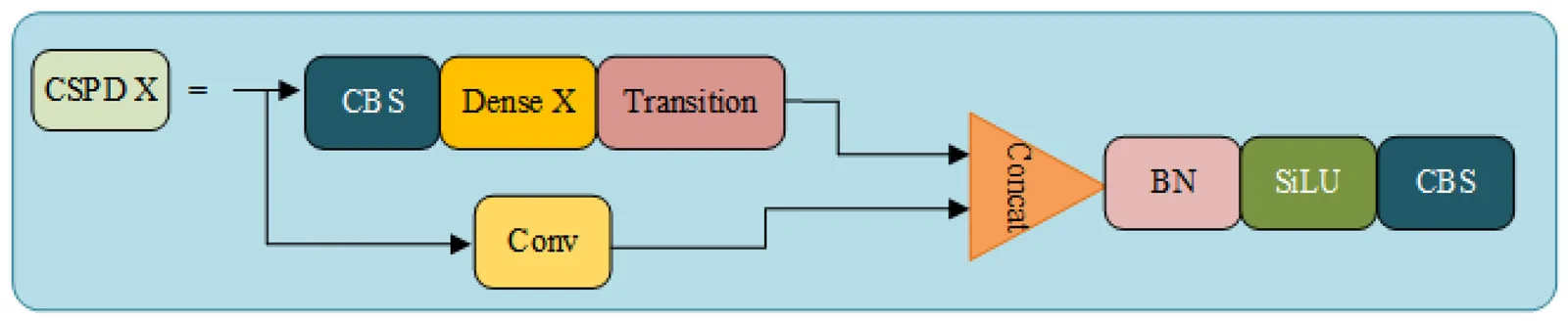
CSPDX structure diagram.

**Figure 7:**
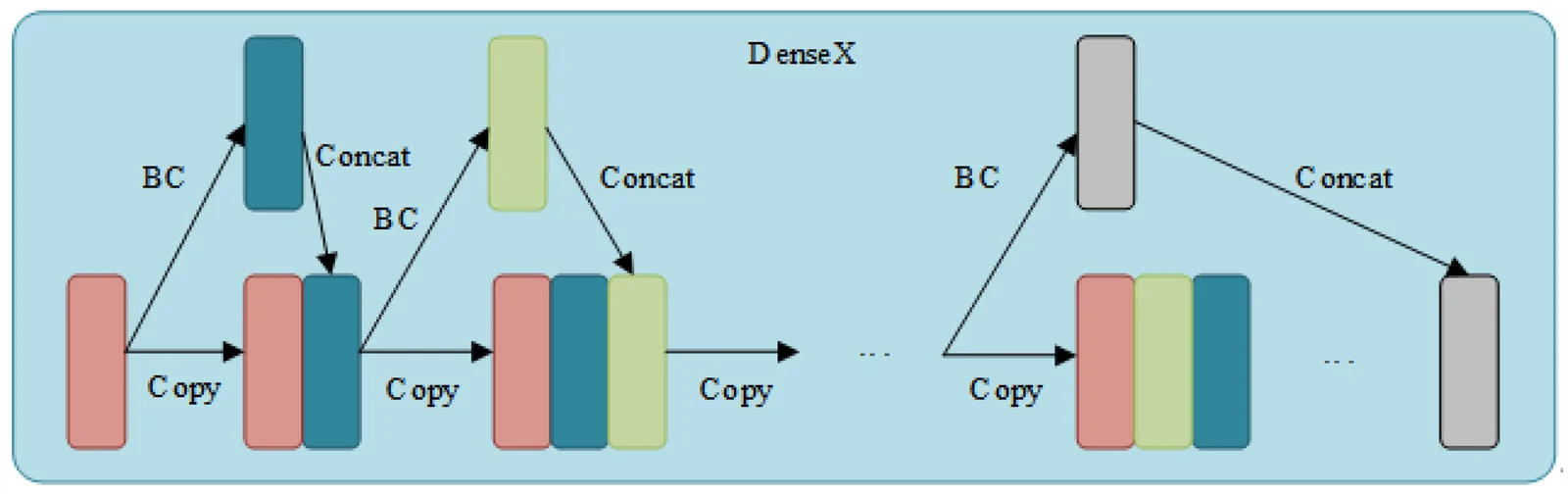
DenseX structure diagram.

The feature extraction unit of CSP-UNet employs CSPDX blocks, which comprise a local residual layer and a local transition layer, to reduce computational redundancy. The detailed structure is shown in Figure 6. In a CSPDX block, the feature map generated in the previous stage is divided into two parts, namely $x_{0}=[x_{0}\prime ,x_{0}\prime \prime ]$. For the two branches $x_{0}\prime$ and $x_{0}\prime \prime$, the former is directly connected to the end of the stage after passing through the transition layer, and the latter is connected to the end of the stage after passing through a dense block of X convolutional blocks and the transition layer, as shown in Figure 7. All steps implemented in a CSPDX block are as follows. The output of the local residual layer $\left[x_{0}\prime \prime ,x_{1},\ldots ,x_{k}\right]$ is passed to a transition layer, and the outputs $X_{T}$ and $X_{T}$ are connected to the output $X_{V}$ of the partial transition layer. Then, the output $X_{U}$ is obtained. The feedforward transfer and weight update steps of a CSPDX block are expressed as shown in Formulas (5) and (6), respectively.

(5) $$\begin{array}{l} x_{k}=w_{k}*[{x_{0}}^{\prime \prime },x_{1},\cdots ,x_{k-1}]\\ x_{T}=w_{T}*[{x_{0}}^{\prime \prime },x_{1},\cdots ,x_{k}]\\ x_{V}=w_{V}*[{x_{0}}^{\prime },{x_{1}}^{\prime }]\\ x_{U}=w_{U}*[x_{V},x_{T}] \end{array}$$

(6) $$\begin{array}{l} {w_{k}}^{\prime }=f(w_{k},{g_{0}}^{\prime \prime },g_{1},\cdots ,g_{k-1})\\ {w_{T}}^{\prime }=f(w_{T},{g_{0}}^{\prime \prime },g_{1},\cdots ,g_{k})\\ {w_{V}}^{\prime }=f(w_{V},{g_{0}}^{\prime },{g_{1}}^{\prime })\\ {w_{U}}^{\prime }=f(w_{U},w_{V},w_{t}) \end{array}$$

Equations (5) and (6) show that the gradient from the local residual layer is calculated separately. Additionally, the feature gradient that does not pass through the residual layer is calculated separately. For the gradient information of updated weights, different feature information does not contain duplicate gradient information from each other.

In addition, a convolution operation with a stride of 2 and a convolution kernel of 3, instead of a max-pooling operation, is used to preserve more characteristic information.

#### 2.3.2. Decoder with a CSP2 Structure

**Figure 8:**
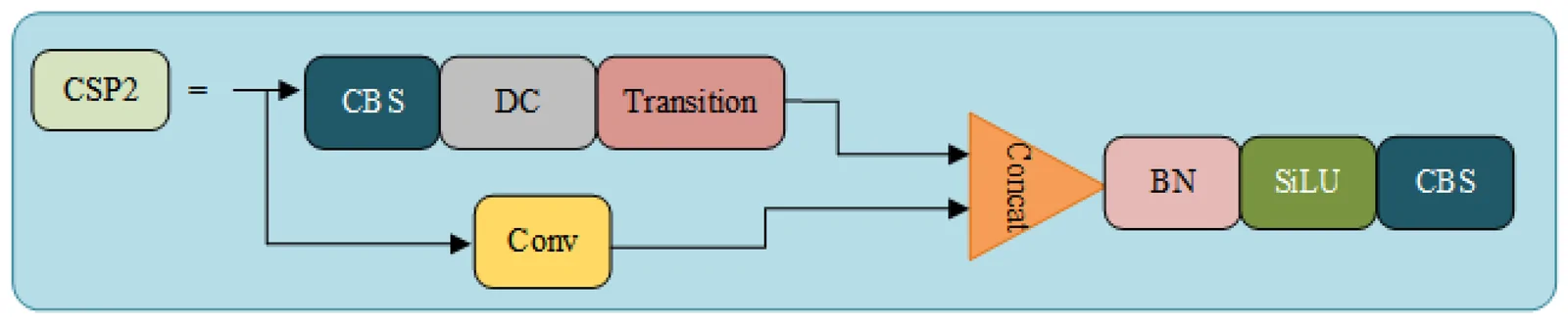
CSP2 structure diagram.

The decoder is a crucial part of the model because it is responsible for feature fusion and upsampling. It aims to restore features while also reducing the loss of spatial information that occurs due to the downsampling process. The feature map from the corresponding encoder stage is integrated with the upsampled feature map based on the CSP2 block, and the double convolution (DC) block structure in the classic UNet decoder is replaced by the CSP2 block. In this way, more low-level semantic information is obtained, which refines the segmentation results. However, this will result in some information redundancy. To reduce the complexity of the decoder, the DenseX block in CSPDX is replaced with a DC block. The remaining components are kept the same as those in CSPDX, resulting in the CSP2 structure. The detailed structure of the CSP2 block is shown in Figure 8. The CSP2 convolution block reduces redundant feature computation in the decoder while maintaining effective feature fusion. This design reduces network complexity while preserving segmentation performance.

The ultimate goal of the segmentation process is to highlight regions with high-quality ROI values. In our approach, we first normalize the segmentation maps produced by CSP-UNet, which results in normalized matrices. These normalized matrices are then multiplied elementwise with the corresponding original matrices. This stepwise process allows us to successfully obtain regions characterized by high-quality ROI values.

#### 2.3.3. Activation Function

**Figure 9:**
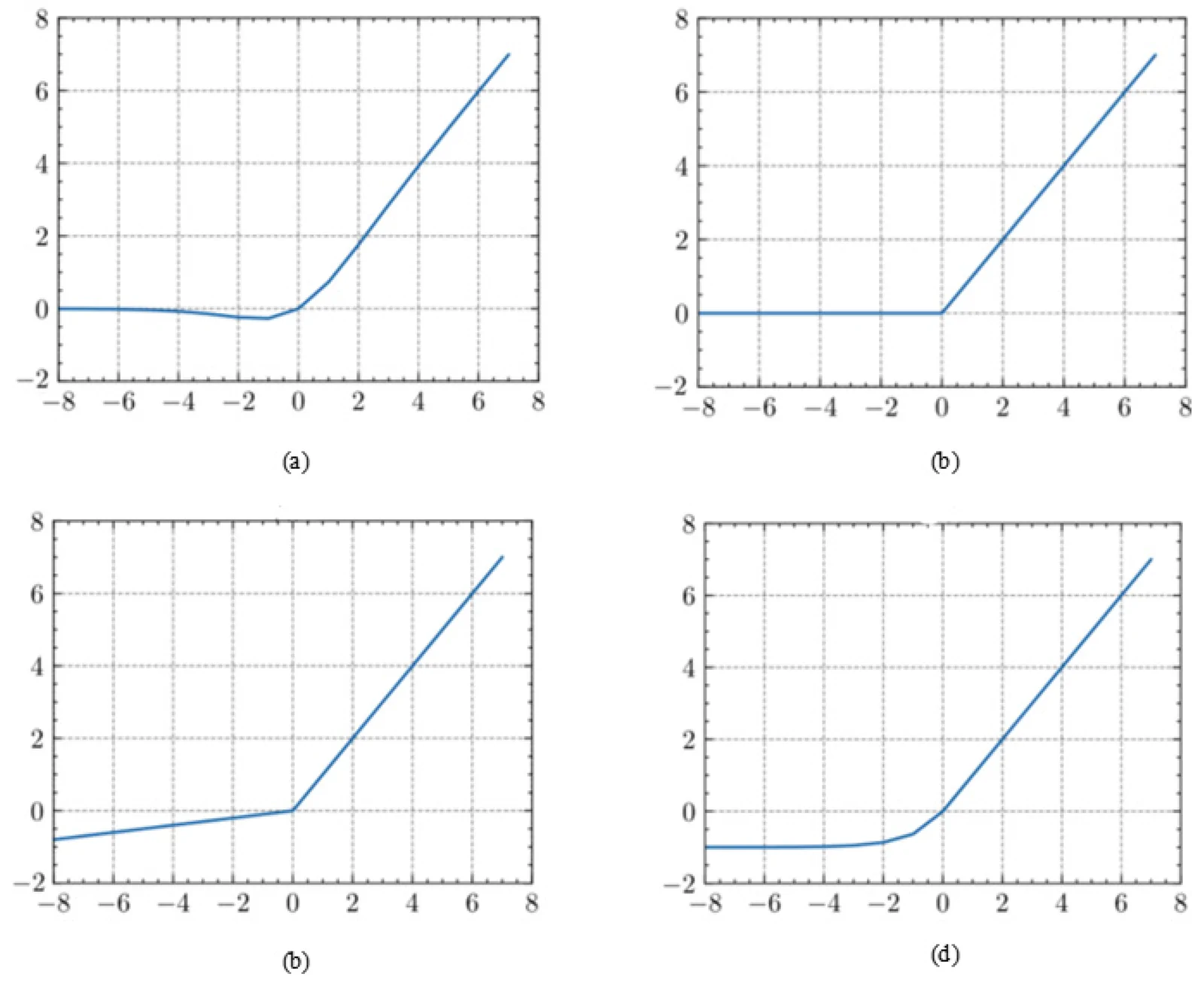
Activate function; (**a**) SiLU, (**b**) Relu, (**c**) Leaky Relu, (**d**) ELU.

The SiLU activation function has self-stabilizing properties due to its smooth formulation and implicit regularization effects, which help prevent excessive weight updates. Due to its smooth and continuously differentiable formulation, SiLU facilitates stable gradient propagation during network optimization and may contribute to improved training stability. In addition, the non-monotonic property of SiLU allows the network to maintain more flexible feature representations and reduce unnecessary weight updates. Compared with Leaky ReLU, ReLU, and ELU activation functions (Figure 9b–d), SiLU is non-monotonic over the entire input range, enabling adaptive adjustment of feature responses according to different input values. Therefore, incorporating the SiLU activation function into CSP-UNet provides more stable nonlinear feature representation and facilitates network optimization. The mathematical formulations of these activation functions are presented in Equations (7)–(10).

(7) $$f(x)=\frac{x}{1+e^{-x}}$$

(8) f(x)=max(0,x)

(9) $$f(x)=\left\{\begin{array}{l} x\;\;\;\;\;\;\;,x>0\\ ax\;\;\;\;\;,x\leq 0 \end{array}\right.$$

(10) $$f(x)=\left\{\begin{array}{l} x\;\;\;\;\;\;\;\;\;\;\;\;\;\;\;\;\;,x>0\\ \alpha (e^{x}-1)\;\;\;\;,x\leq 0 \end{array}\right.$$

#### 2.3.4. Loss Function

The output of the network is a two-channel image, with each pixel assigned a probability value indicating its association with either the foreground (hand tissue) or background (noise). The foreground comprises only one-third or less of the background in the experimental dataset, leading to a bias toward the background and resulting in under-segmentation when identifying the concave surface of finger seams. During training, the objective function combines cross-entropy loss and Dice loss. Cross-entropy loss provides pixel-wise classification supervision, while Dice loss is introduced to alleviate the influence of class imbalance between the hand region and the background. The Dice loss is defined in Equation (11), and its smoothed form is given in Equation (12).

(11) $$L_{dice}=1-\frac{2\sum_{x\in \Omega }p_{l}(x)g_{l}(x)}{\sum_{x\in \Omega }p_{l}^{2}(x)+\sum_{x\in \Omega }g_{l}^{2}(x)}$$

(12) $$L_{dice}=1-\frac{2\sum_{x\in \Omega }p_{l}(x)g_{l}(x)+1}{\sum_{x\in \Omega }p_{l}^{2}(x)+\sum_{x\in \Omega }g_{l}^{2}(x)+1}$$

where $p_{l}(x)$ is the probability that the pixel belongs to the background and $g_{l}(x)$ is the probability that the pixel belongs to the foreground.

### 2.4. Lightweight Pruning of the CSP-UNet Structure

**Figure 10:**
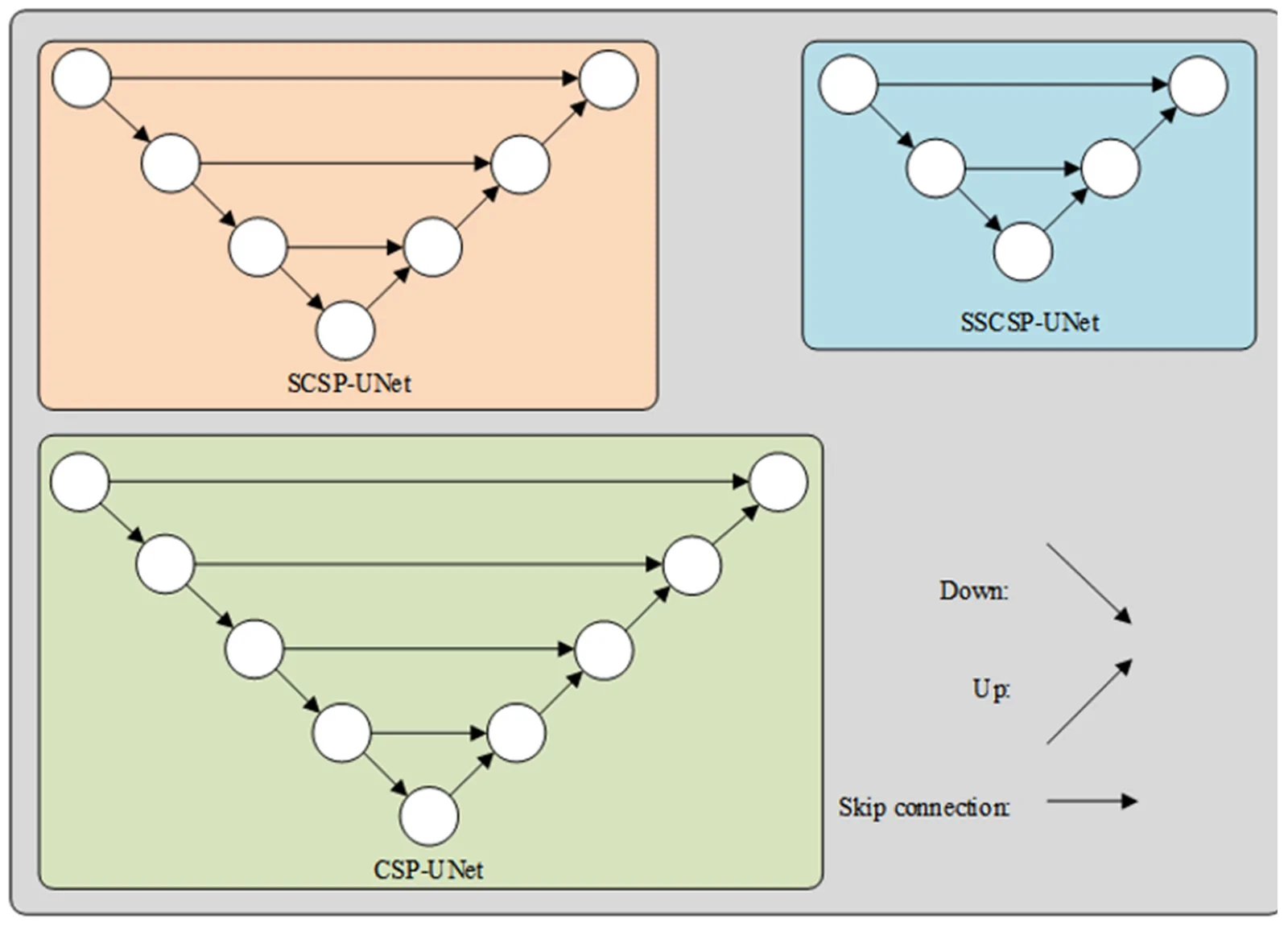
Lightweight pruning CSP-UNet structure diagram.

The model can be pruned to obtain a smaller network in situations that do not require high segmentation accuracy, such as those involving mobile devices. A lightweight CSP-UNet called SCSP-UNet with reduced numbers of downsampling and upsampling blocks in the final stage of the network is proposed, and the performance of SCSP-UNet is validated and compared with that of a benchmark network. SSCSP-UNet is the ultimate verification network for pruned networks. The explored model structures are shown in Figure 10.

### 2.5. Evaluation Criteria and Experimental Platform

The Dice score (Dice), pixel accuracy (PA), mean pixel accuracy (MPA), mean intersection over union (MIoU) and model size (Parameters) can be used to evaluate the performance of a model. When using these evaluation criteria, a confusion matrix needs to be introduced. We assume that there must be k + 1 classes (including k target classes and one background class), and $p_{ij}$ represents the total number of pixels that belong to class i but are predicted to be class j. Specifically, $p_{ii}$ represents true positives, $p_{ij}$ represents false positives, and $p_{ji}$ represents false negatives.

Pixel accuracy is the percentage of pixels correctly labelled as a percentage of the total number of pixels, as shown in Formula (13):

(13) $$PA=\frac{\sum_{i=0}^{k}p_{ii}}{\sum_{i=0}^{k}\sum_{j=0}^{k}p_{ij}}$$

The mean pixel accuracy for a category is obtained by calculating the ratio of correctly classified pixels within the category to the total number of pixels in the class and then taking the average of these ratios, as shown in Formula (14).

(14) $$MPA=\frac{1}{k+1}\sum_{i=0}^{k}\frac{p_{ii}}{\sum_{j=0}^{k}p_{ij}}$$

The class-average intersection ratio is the proportion of correctly classified pixels for each class, as defined in Formula (15).

(15) $$MIoU=\frac{1}{k+1}\sum_{i=0}^{k}\frac{p_{ii}}{\sum_{j=0}^{k}p_{ij}+\sum_{j=0}^{k}p_{ji}-p_{ii}}$$

In Formula (15), when i=m, $p_{mm}$ represents true positives, $\sum_{i=0}^{k}p_{ii}$ is the number of pixels in category m but predicted to fall within an another class, and $\sum_{j=0}^{k}p_{ji}$ represents the number of pixels in other classes but are predicted to fall within category m.

All experiments were performed on a single platform, as shown in Table 2.

To ensure a fair comparison of experimental results and account for GPU memory and gradient stability requirements, all input images were normalized to 256 × 256 × 1. During training, CSP-UNet, Res-UNet, Attention U-Net, Swin U-Net, and Classic U-Net were trained for 500 epochs with a batch size of 2 under the same experimental settings. Considering the available GPU memory and the small batch size, the initial learning rate was set to 0.00001 to maintain stable optimization. The RMSprop optimizer was used with a weight decay of 1 × 10^−8^ and a momentum of 0.9. The learning rate was adaptively adjusted using a ReduceLROnPlateau (PyTorch, Meta AI, Menlo Park, CA, USA) scheduler according to the validation Dice score, with a patience of 2. The training objective combined cross-entropy loss and Dice loss. These settings were kept consistent across the compared networks to ensure a fair and reproducible comparison.

## 3. Results

### 3.1. The Adaptive Gaussian Histogram Matching Results

**Figure 11:**
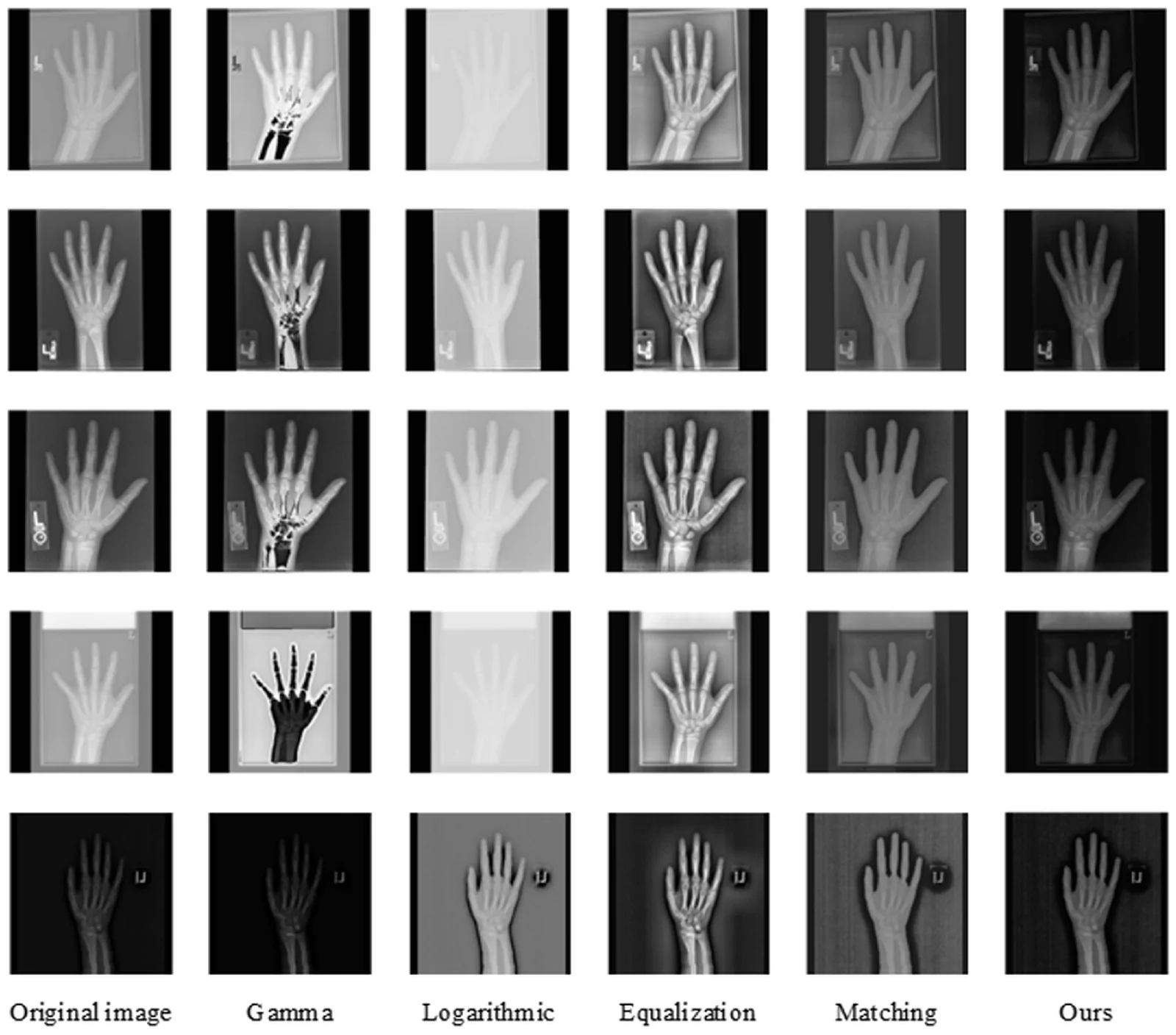
Segmentation results with different preprocessing strategies.

The adaptive Gaussian histogram-matching method for hand X-ray image preprocessing is promising. Compared with traditional methods such as gamma transform, logarithmic transform, histogram equalization and histogram matching, this method has higher flexibility in the preprocessing of X-ray images, and the processed images are used for semantic segmentation. Additionally, overall, this approach performs better than traditional methods. For comparison, five images with different brightness levels, content complexities, and IOU ratios were randomly selected for processing with different methods. Figure 11 shows the performance of data enhancement with the adaptive Gaussian histogram-matching approach compared to that achieved with other data enhancement methods.

The influence of different grayscale preprocessing strategies, including gamma transformation, logarithmic transformation, histogram equalization, histogram matching, and adaptive Gaussian histogram matching, was evaluated using 500 randomly selected images. This experiment was designed to compare the relative effectiveness of different preprocessing methods under the same segmentation framework rather than to provide an independent evaluation of model generalization. As shown in Table 3, the adaptive Gaussian histogram-matching strategy achieved the highest Dice score, PA, MPA, and mIoU among the evaluated preprocessing methods, indicating that it provided better segmentation performance under the evaluated conditions. Compared with the original, gamma-transformed, logarithmically transformed, histogram-equalized, and histogram-matched images, the adaptive Gaussian histogram-matching strategy improved the Dice score by 14.11%, 33.37%, 17.66%, 0.52%, and 1.11%, respectively.

### 3.2. CSP-UNet Results

**Figure 12:**
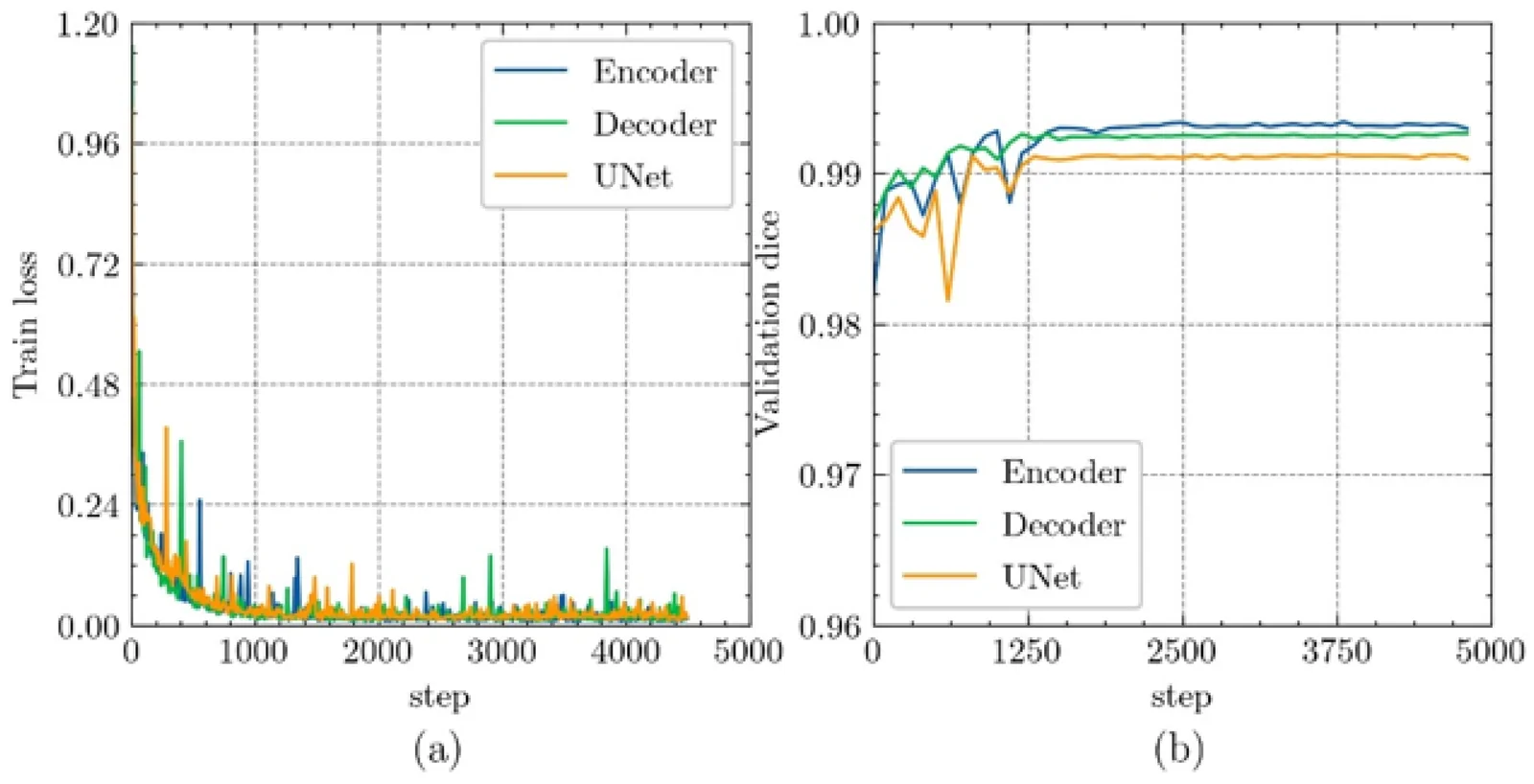
The training and validation process of the encoder and decoder ablation experiments: (**a**) train loss; (**b**) validation Dice.

This section investigates the individual and combined contributions of the CSPDX encoder and CSP2 decoder through ablation experiments. As illustrated in Table 4, under identical experimental settings, the CSPDX-based encoder and CSP2-based decoder reduce the number of parameters by 28.36% and 42.55%, respectively, compared with the vanilla U-Net. However, individual replacement of either the encoder or decoder does not improve segmentation accuracy compared with the baseline U-Net. These two designs mainly contribute to reducing model complexity while maintaining comparable segmentation capability. The training convergence curves are visualized in Figure 12a,b. The networks integrated with CSPDX and CSP2 modules exhibit similar convergence trends to those of vanilla U-Net. When both modules were incorporated, the complete CSP-UNet achieved a Dice score of 0.9927 with 20.01 M parameters under the current experimental setting. However, because the individual replacement of either CSPDX or CSP2 did not improve the Dice score over the U-Net baseline, the observed performance of the complete architecture should not be interpreted as evidence of an independent performance gain from either module. Instead, the ablation results mainly demonstrate that the combined architecture substantially reduces the parameter count while maintaining segmentation performance comparable to that of U-Net.

**Figure 13:**
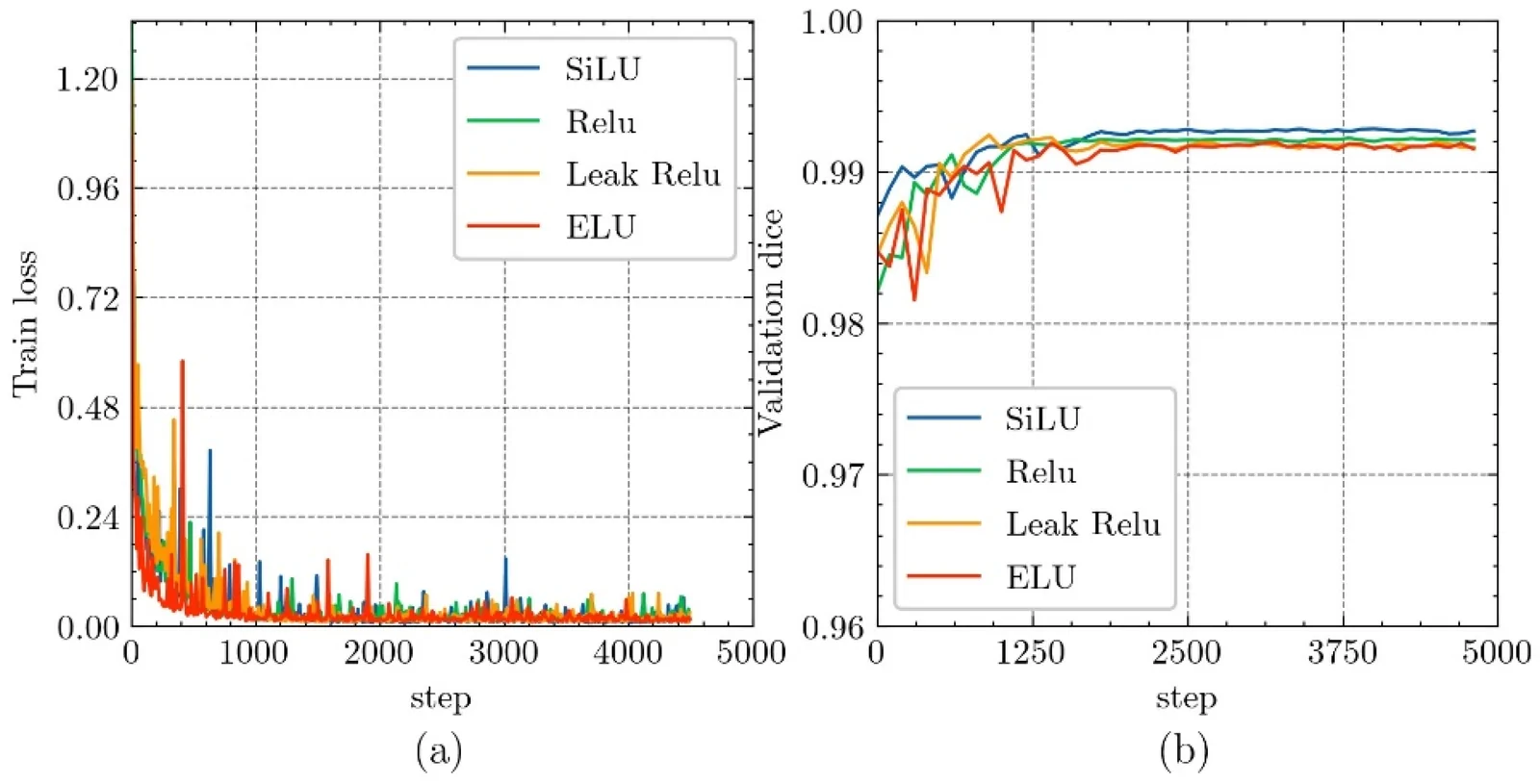
Training and validation curves obtained using different activation functions: (**a**) training loss and (**b**) validation Dice.

The performance of various activation functions in CSP-UNet, such as ReLU, leaky ReLU, ELU, and SiLU functions, was assessed for hand X-ray image segmentation. Metrics such as training loss and the verification Dice score were utilized during the experiments. Figure 13a displays the training loss of CSP-UNet with different activation functions, and Figure 13b presents the validation Dice scores obtained using different activation functions. Under the current experimental setting, the model using the SiLU activation function exhibited relatively stable training behavior and achieved a competitive validation Dice score among the evaluated activation functions.

### 3.3. Results of Different Segmentation Models

**Figure 14:**
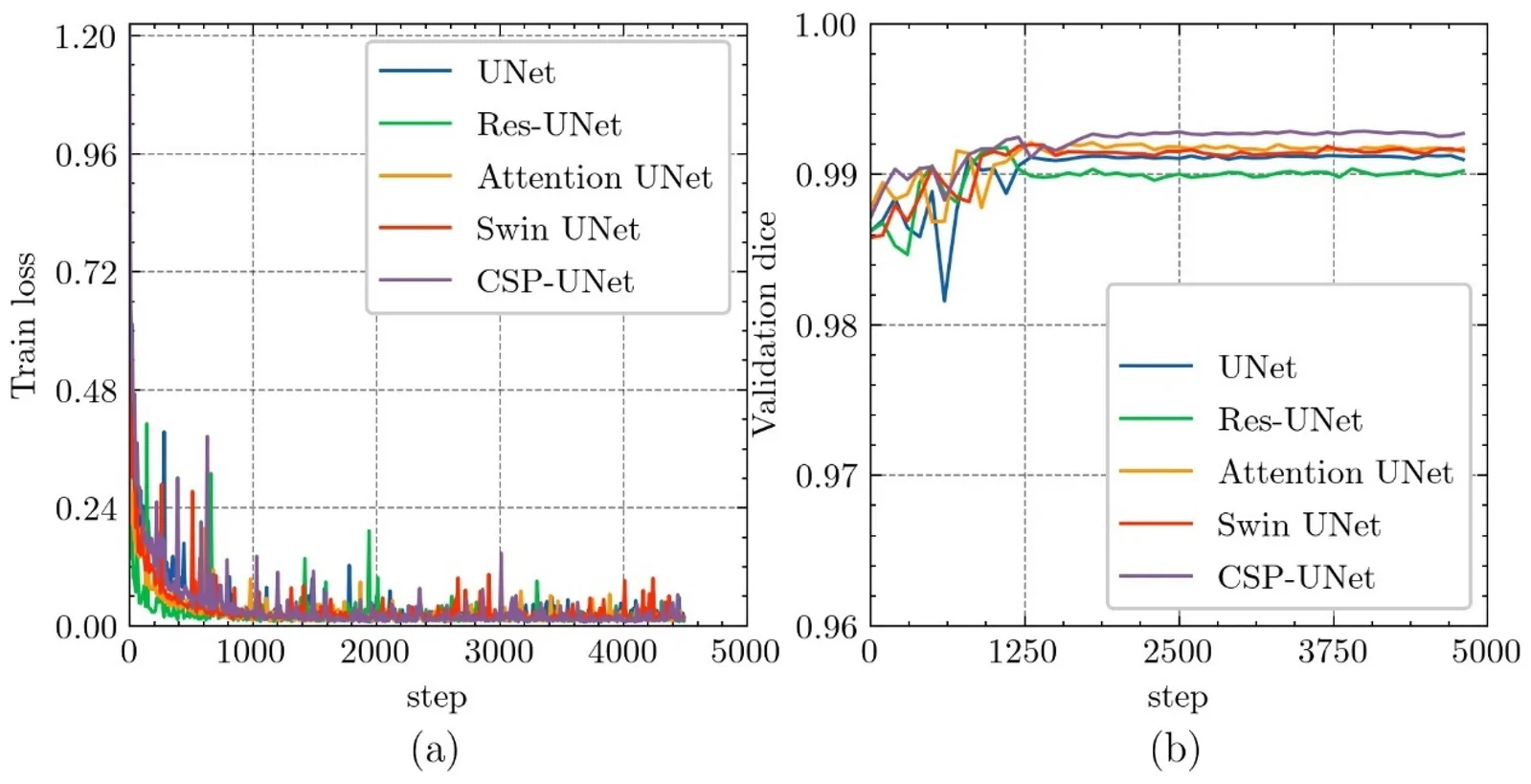
The training and validation process of different segmentation networks: (**a**) train loss; (**b**) validation Dice.

Figure 14a,b show the training and validation curves of different U-shaped networks for hand X-ray image segmentation. Under the current experimental setting, CSP-UNet exhibited a relatively rapid increase in validation Dice during the early training stage and subsequently converged to a level comparable to those of the other networks. Because these curves were obtained from a single fixed dataset split, they describe only the convergence behavior observed under the present experimental conditions.

**Figure 15:**
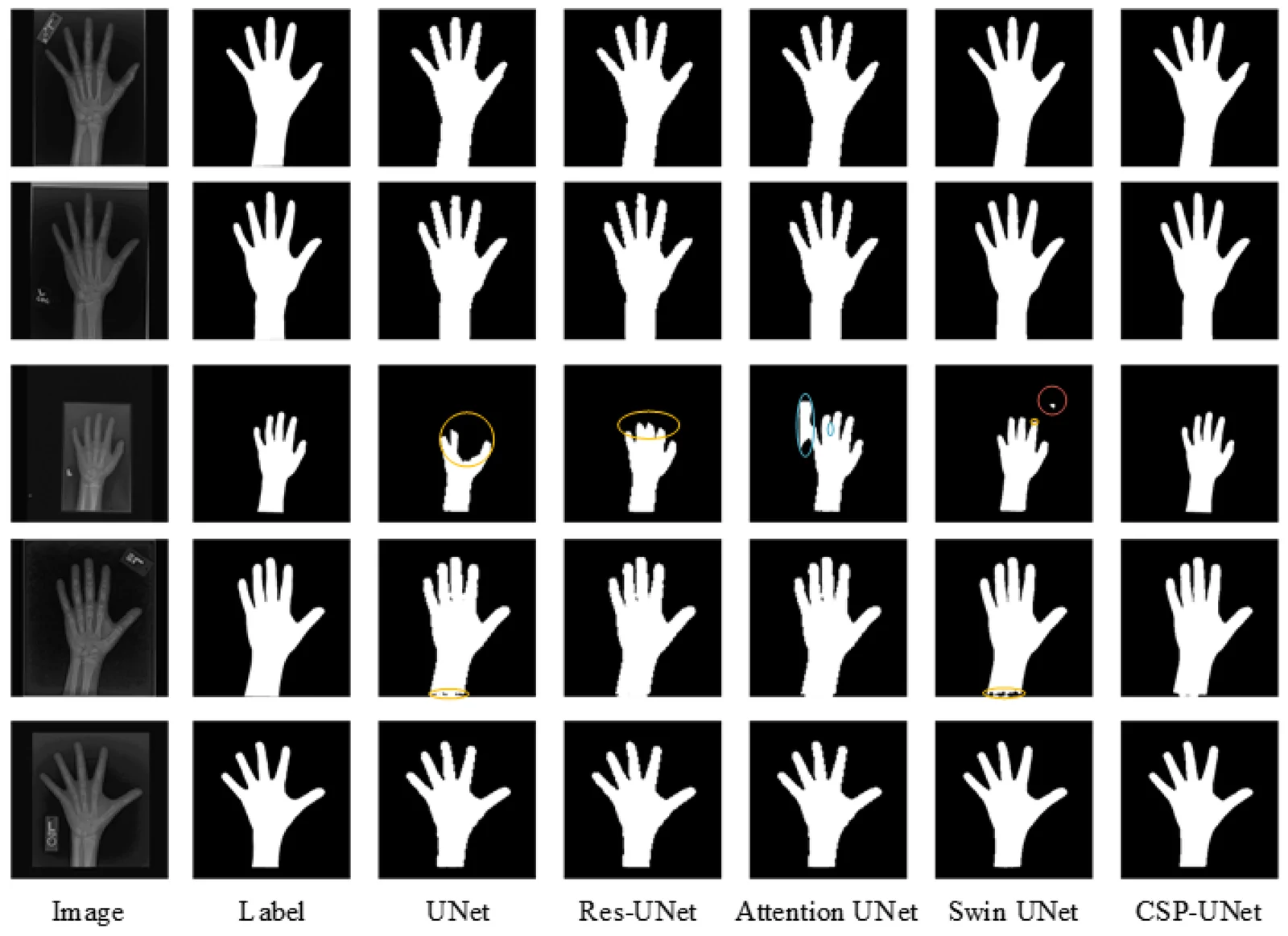
Qualitative comparison of segmentation results obtained by different networks. Blue circles indicate over-segmentation, yellow circles indicate under-segmentation, and red circles indicate incorrect segmentation.

The quantitative comparison of different segmentation networks is presented in Table 5. CSP-UNet achieved a Dice score of 0.9927, PA of 0.9839, MPA of 0.9810, and mIoU of 0.9542. Although the improvements in segmentation performance over the compared networks are relatively limited, CSP-UNet requires only 20.01 M parameters and 46.43 G FLOPs. The parameter count of CSP-UNet is substantially reduced compared with conventional U-shaped networks, while its FLOPs remain within a comparable computational range. Specifically, CSP-UNet requires only 20.01 M parameters and 46.43 G FLOPs, while maintaining comparable segmentation performance. These results indicate that CSP-UNet reduces model complexity while maintaining comparable segmentation performance. The segmentation results in Figure 15 illustrate instances of over-segmentation (circled in blue), under-segmentation (circled in yellow), and segmentation errors (circled in red). In relatively complex scenes, Classic U-Net and Res-UNet exhibit evident over-segmentation, while Attention U-Net shows under-segmentation and Swin U-Net presents segmentation errors in some regions. In comparison, CSP-UNet provides visually comparable segmentation results across these examples while using substantially fewer model parameters.

### 3.4. Lightweight CSP-UNet Segmentation Experiment

**Figure 16:**
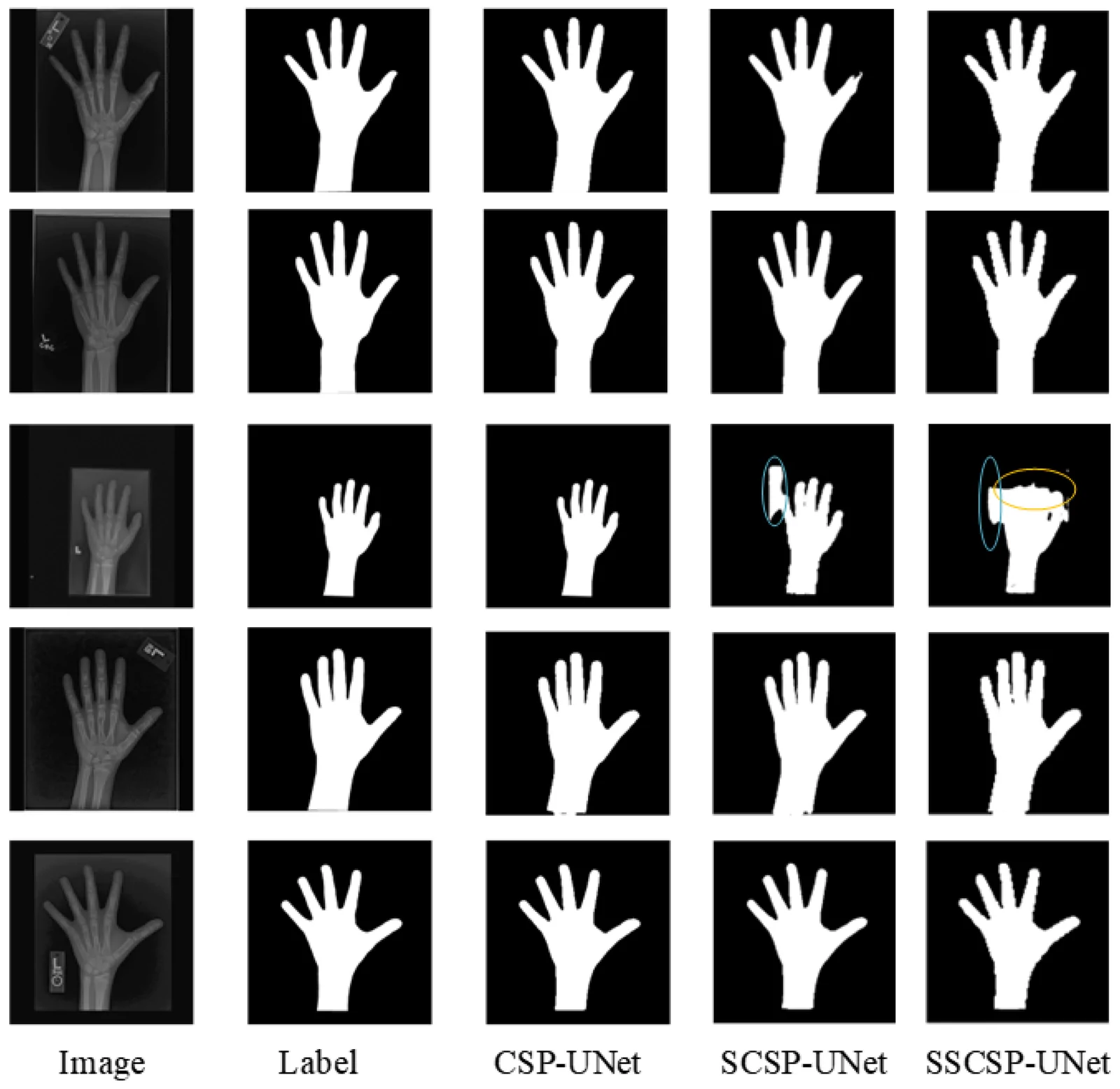
Test results of different lightweight CSP-Unet. (Note: Blue areas denote over-segmentation regions, and yellow areas denote under-segmentation regions.)

**Figure 17:**
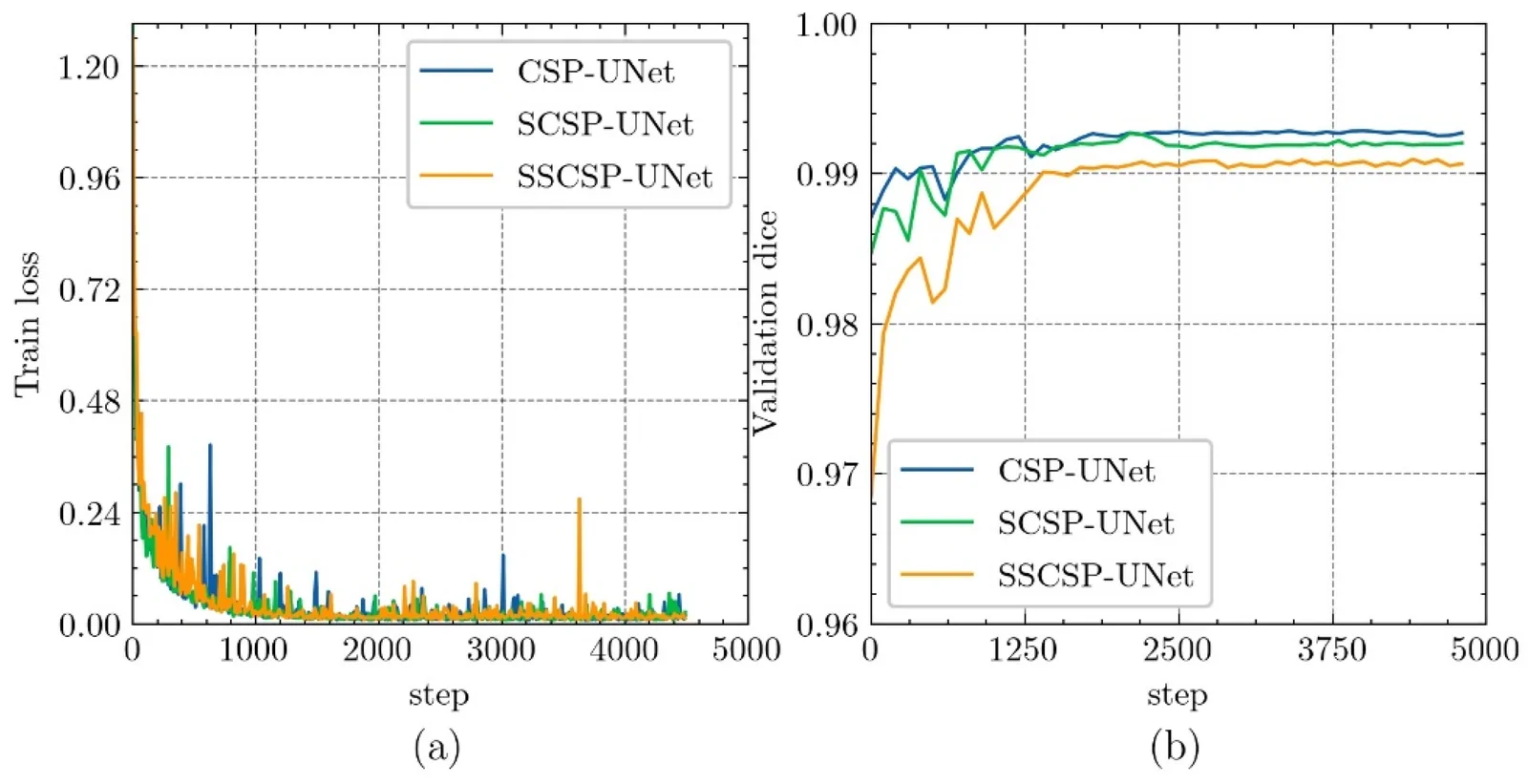
The training and validation process of different lightweight CSP-UNet: (**a**) train loss; (**b**) validation Dice.

Figure 16 illustrates the segmentation results of the lightweight variants SCSP-UNet and SSCSP-UNet, with over-segmentation areas marked in blue and under-segmentation areas marked in yellow. Reducing the number of upsampling and downsampling blocks decreases model complexity but may reduce the ability of the network to preserve fine boundary information, resulting in occasional over-segmentation and under-segmentation. This observation indicates a trade-off between model compactness and segmentation accuracy. Therefore, SCSP-UNet is more suitable for resource-constrained scenarios where model efficiency is prioritized, whereas CSP-UNet remains preferable when higher segmentation accuracy is required. Figure 17a,b show the training loss and validation Dice scores of the lightweight CSP-UNet variants, respectively.

The segmentation performance of the CSP-UNet structure with a reduced number of upsampling and downsampling blocks is shown in Table 6. Based on metrics such as Dice scores, PA, MPA, MIoU, and the number of parameters, the lightweight CSP-UNet demonstrated acceptable performance in hand X-ray image segmentation. As shown in Table 6, the number of parameters in SCSP-UNet represents only 29.99% of those in CSP-UNet and a mere 8.68% of those in UNet. To assess the limitations of the pruning process for CSP-UNet, we examined the impact of reducing the number of up-sample and down-sample blocks in the network, resulting in the creation of SSCSP-UNet. The parameter count of SSCSP-UNet is 98.33% of that of SCSP-UNet. Although SSCSP-UNet further reduces the parameter count from 6.0 M to 5.9 M, its Dice score decreases from 0.9912 to 0.9873, indicating that further structural simplification begins to compromise segmentation performance. Consequently, the parameter count of SCSP-UNet represents the minimum for CSP-UNet post-pruning, ensuring acceptable performance in hand X-ray image segmentation.

## 4. Discussion

The adaptive Gaussian histogram-matching strategy improved the consistency of grayscale distributions among hand X-ray images by generating a dataset-specific matching template through K-means clustering. Compared with the original images and those processed using gamma transformation, logarithmic transformation, histogram equalization, and conventional histogram matching, the proposed preprocessing method improved the Dice score under the evaluated segmentation framework by 14.11%, 33.37%, 17.66%, 0.52%, and 1.11%, respectively. These results suggest that the proposed preprocessing strategy can improve segmentation performance under the current experimental conditions. In addition, integrating CSPDX and CSP2 blocks into the complete encoder–decoder architecture resulted in CSP-UNet achieving a Dice score of 0.9927 with only 20.01 M parameters. The ablation results indicate that individual CSPDX or CSP2 replacement does not improve the Dice score over the U-Net baseline, whereas the complete CSP-UNet achieved a Dice score of 0.9927 with 20.01 M parameters under the current experimental setting. The parameter count of CSP-UNet corresponds to 28.95% of Classic U-Net, 20.52% of Res-UNet, 28.63% of Attention U-Net, and 18.09% of Swin U-Net, indicating that the primary advantage of CSP-UNet lies in reducing model complexity while maintaining comparable segmentation performance. The further-pruned SCSP-UNet retained a Dice score of 0.9912 with only 6 M parameters, representing a 0.15% decrease compared with CSP-UNet while reducing the parameter count to 29.99% of CSP-UNet. This result demonstrates the trade-off between model compactness and segmentation performance. Although CSP-UNet achieved comparable segmentation performance, several limitations should be acknowledged. First, the model was evaluated using a single fixed training-validation-test split, and the stability of performance under different data partitions was not further investigated. Second, independent external validation using additional datasets or multi-center data was not performed. Third, the statistical significance of the small performance differences among compared models was not evaluated. Therefore, the reported results should be interpreted within the current experimental setting. Future studies will investigate repeated experiments, cross-validation, external validation, and statistical analysis to further assess model robustness and reliability.

## 5. Conclusions

This study proposed CSP-UNet, a lightweight network for hand X-ray image segmentation, together with an adaptive Gaussian histogram-matching method for reducing grayscale distribution differences among input images. The proposed preprocessing strategy improved image intensity consistency, while the CSPDX encoder and CSP2 decoder reduced model complexity while maintaining comparable segmentation performance. CSP-UNet achieved a Dice score of 0.9927 with 20.01 M parameters, and the lightweight SCSP-UNet further reduced the parameter count to 6 M while maintaining a Dice score of 0.9912. Under the current experimental setting, the proposed networks achieved comparable segmentation performance with substantially fewer parameters than the evaluated U-shaped networks. The generated hand-region masks may provide a preprocessing basis for subsequent quantitative analysis of hand X-ray images. However, the current study was conducted on a single hand X-ray segmentation task without external validation or statistical significance testing. Future work will therefore focus on multi-center and multi-dataset validation, repeated experiments or cross-validation, statistical significance analysis, and further evaluation of model robustness under variations in image quality and acquisition conditions.

## Figures and Tables

**Table 1:** Convolution parameters in the structure of CSP-Unet.

Part	Filters	Filter Size	Output Shape
DC	64	3 × 3	(256,256,64)
Down	64	3 × 3	(256,256,64)
CSPD2	128	3 × 3	(128,128,128)
Down	128	3 × 3	(128,128,128)
CSPD4	256	3 × 3	(64,64,256)
Down	256	3 × 3	(64,64,256)
CSPD8	512	3 × 3	(32,32,512)
Down	512	3 × 3	(32,32,512)
CSPD8	512	3 × 3	(16,16,512)
UP	512	3 × 3	(32,32,512)
CSP2	512	3 × 3	(32,32,512)
UP	256	3 × 3	(64,64,256)
CSP2	256	3 × 3	(64,64,256)
UP	128	3 × 3	(128,128,128)
CSP2	128	3 × 3	(128,128,128)
UP	64	3 × 3	(256,256,64)
CSP2	64	3 × 3	(256,256,64)
OC	2	3 × 3	(256,256,2)

**Table 2:** Experimental platform parameters.

Experiment	Model
Platform	Ubuntu 20.04
CPU	Intel(R) Core(TM) i7-10875H(Intel Corporation, Santa Clara, CA, USA)
GPU	GeForce RTX 2060(NVIDIA Corporation, Santa Clara, CA, USA)
System memory	16 GiB
SSD	Samsung SSD 970 EVO Plus 1TB (Samsung Electronics Co., Ltd., Suwon, South Korea)
Frame	Pytorch (version 2.0.1)
Language	Python (version 3.8)

**Table 3:** The effect of different data enhancement on segmentation.

Project	Original Image	Gamma	Logarithmic	Equalization	Matching	Ours
Dice	0.8605	0.7362	0.8345	0.9768	0.9711	0.9819
PA	0.8560	0.7160	0.8292	0.9582	0.9472	0.9671
MPA	0.7274	0.6542	0.6925	0.9563	0.9391	0.9631
MIoU	0.6310	0.5963	0.6132	0.8957	0.8425	0.9085

**Table 4:** Ablation study of the CSPDX encoder and CSP2 decoder.

Model	CSPDX Encoder	CSP2 Decoder	Dice	PA	MPA	Parameters
U-Net	×	×	0.9910	0.9793	0.9618	69.10 M
U-Net + CSPDX	√	×	0.9876	0.9813	0.9610	49.50 M
U-Net + CSP2	×	√	0.9867	0.9828	0.9592	39.70 M
CSP-UNet	√	√	0.9927	0.9839	0.9810	20.01 M

(Note: √ indicates satisfied, × indicates unsatisfied).

**Table 5:** Quantitative comparison of different segmentation networks.

Project	UNet	Res-UNet	Attention UNet	Swin UNet	CSP-UNet
Dice	0.9910	0.9902	0.9917	0.9914	0.9927
PA	0.9793	0.9824	0.9821	0.9804	0.9839
MPA	0.9618	0.9766	0.9794	0.9651	0.9810
MIoU	0.9318	0.9467	0.9466	0.9322	0.9542
FLOPs	61.43	86.54	63.12	12.24	46.43
Parameters	69.10 M	97.50 M	69.90 M	110.60 M	20.01 M

Note: FLOPs were calculated using the THOP tool (Ultralytics, Los Angeles, CA, USA)with a unified input resolution of 256 × 256. Due to differences in network architectures and implementation details, FLOPs values represent theoretical computational complexity and may not fully reflect practical inference efficiency.

**Table 6:** Lightweight CSP-Unet experiment results.

Project	CSP-UNet	SCSP-UNet	SSCSP-UNet
Dice	0.9927	0.9912	0.9873
PA	0.9839	0.9835	0.9770
MPA	0.9810	0.9817	0.9752
MIoU	0.9542	0.9543	0.9382
Parameters	20.01 M	6 M	5.9 M

## Data Availability

The clinical hand X-ray images collected from the participating hospital are not publicly available because they contain sensitive medical information and are subject to patient privacy, ethical, and institutional restrictions. Access to these data may be considered upon reasonable request to the corresponding author and with permission from the data-providing institution, subject to applicable ethical and data protection requirements.
